# Alleviating academic stress and enhancing interdisciplinary self-efficacy in university libraries: an exploratory mixed-methods study on restorative built-environment interventions

**DOI:** 10.3389/fpsyg.2026.1904029

**Published:** 2026-07-30

**Authors:** Yu Chen, Kai Chen, Yanxia Lyu, Ruicheng Qian

**Affiliations:** 1 Library, Northeastern University at Qinhuangdao, Qinhuangdao, China; 2 Northeastern University at Qinhuangdao, Qinhuangdao, China; 3 School of Business Administration, Northeastern University, Shenyang, China

**Keywords:** academic stress, cognitive decoupling, Double Machine Learning (DML), embodied cognition, Future Learning Center, Liquid Capability (LCI), place attachment, university library

## Abstract

**Background:**

In the digital intelligence era, higher education students—especially those in STEM (Science, Technology, Engineering, and Mathematics) fields—face widespread academic stress, cognitive rigidity, and negative emotional rumination. Traditional psychological interventions are often confined to clinical counseling and questionnaire screenings, which can easily induce a sense of stigma and pathological labeling. Exploring non-invasive, routine physical built-environment interventions is therefore of significant academic value and practical urgency. As a campus “third space,” the university library possesses unique physical and cultural advantages for building a non-evaluative “psychological safety sandbox.”

**Methods:**

This study adopted an exploratory sequential mixed-methods design, utilizing the Library of Northeastern University at Qinhuangdao (a collaborative innovation node of the Future Learning Center Construction across five provinces) as the empirical field. We conducted a longitudinal tracking study from Fall 2025 to Spring 2026, analyzing the behavior database of 871 registered students (1,493 activity records) and focusing on a cohort of *N* = 122 subjects with complete behavioral and psychological tracking data. Quantitative data were obtained via the self-developed “Library Restorative Experience and Capability Questionnaire” (LRECQ). Qualitative text analysis was conducted on students' reflective diaries using thematic analysis.

**Results:**

An independent-samples *t-*test indicated that multimodal interventions combining “static support” (quiet, biophilic study pods) and “dynamic interaction” (embodied handicrafts and physical exercise check-ins) significantly increased students' continuous focused reading time, showing a dramatic expansion compared to the pre-test control group (228.49 ± 75.58 min vs. 86.80 ± 95.56 min, *t*(116) = 8.98, *p* < 0.001). Reliability testing demonstrated that the LRECQ scale has excellent reliability, extracting two high-order “Liquid Capability” (LCI) factors: “Cognitive Liquefaction and Interdisciplinary Reorganization” (LCI-Cognitive, /Cronbach's α = 0.7240) and “Social Collaboration and Organizational Efficacy” (LCI-Social, Cronbach's α = 0.8500). Thematic analysis revealed that weakly correlated sensory-motor interactions occupied working memory resources, triggering “cognitive decoupling” and interrupting DMN-based stress loops. Furthermore, applying abstract academic schemas to culturally warm objects (“schema adaptation”) dissolved the temporal distortion of the “forever fallacy,” sparking a surge in adaptive self-efficacy.

**Conclusion:**

This study confirms that university libraries can function as multimodal built-environment restorative platforms through two empirically validated mechanisms—sensory-motor cognitive decoupling (weakly correlated manual/physical tasks preempt working memory, severing the Default Mode Network from academic rumination loops) and /Cronbach's (abstract professional knowledge liquefies into creative agency when applied in non-evaluative, culturally warm contexts)—together forming a dual-phase restoration-activation cycle. Three generalizable regularities are identified: (1) the sensory-motor interrupt as a universal, non-stigmatizing pattern-break principle; (2) non-evaluative schema liquefaction as a cross-domain capability-building regularity; and (3) a ~23-25% voluntary-sample selection bias consistently inflating naive effect estimates, correctable by DML. These findings provide a theoretically grounded, data-evidenced paradigm for non-invasive campus mental health space governance.

## 1 Introduction

### 1.1 Systematic roots of college student mental health crisis and the urgency of non-invasive interventions

While generative artificial intelligence (AIGC) and large language models (LLMs) (Carlsson, 2026; Wang et al., 2023; Bancroft and Rogers, 2024; Hou et al., 2023) are rapidly reshaping the information ecology of higher education, university students are falling into a modern dilemma of “knowledge suspension” and “academic stress.” High-intensity GPA evaluations, heavy course loads, and future employment uncertainties continuously drain college students' psychological energy, leading to widespread anxiety, depression, and obsessive cognitive rumination (Bladek, 2021; Jiang, 2025). Traditional crisis interventions often carry a stigma of pathological labeling (Cox and Brewster, 2020; Hammock et al., 2023). Therefore, exploring a non-invasive, routine physical built-environment and behavioral regulation scheme in daily campus life to block negative emotions is a major issue to be solved in higher education administration and positive psychology (Guo, 2025; Zhang, 2021).

### 1.2 Restorative environment theory and the “psychological safety sandbox” attribute of university libraries

Classic research in environmental psychology indicates that an individual's psychological state is in deep resonance and interaction with their physical space. The Attention Restoration Theory (ART) proposed by Kaplan suggests that long-term high-intensity directed attention leads to prefrontal cortex fatigue. In contrast, restorative environments rich in natural elements, possessing a sense of “being away,” “extent,” “fascination,” and “compatibility,” can spontaneously activate involuntary attention, thereby achieving rapid recovery of directed attention and reducing psychological entropy (Cox, 2019; Zuo and Chi, 2024). Ulrich's Stress Recovery Theory (SRT) also points out that specific physical landscapes and spatial perceptions can regulate autonomic arousal in the central nervous system, reducing physiological cortisol levels (Li and Wang, 2025; Gillis and Gatersleben, 2015).

In the overall layout of university campuses, the library serves as a “third space” distinct from dormitories and classrooms—a quiet, highly inclusive, and non-evaluative public building free of GPA and academic assessment pressures (Zhang, 2024; Crook and Mitchell, 2012). This unique physical and cultural attribute endows it with the ideal potential to construct a “psychological safety sandbox” (Liu et al., 2025; Yuan, 2020). The library should not only be the academic foundation for students' knowledge discovery but also transform into a “therapeutic landscape” providing long-term psychological absorption and emotional buffering (Zhang and Zhang, 2026; Gan, 2025). This study explores how spatial physical environments and interaction mechanisms can activate place attachment systems (PAS) to provide gentle comfort against academic stress.

### 1.3 The “cognitive decoupling” hypothesis in weakly correlated sensory-motor interactions

In exploring the specific effects of library space interventions, this study proposes a dual complementary path:

- “Static Support” relies on quiet physical spaces, curated therapeutic reading, and mental immersion, emphasizing inward-oriented directed attention recovery and physiological arousal tuning (Lei, 2025; Huang et al., 2026).
- “Dynamic Interaction” utilizes embodied labor in collaborative spaces (such as intangible cultural heritage handicrafts, e.g., filigree enamel, lacquer fans, paper cutting, and braiding) and sports check-ins (e.g., the “Bookworm Games”), emphasizing active behavior activation (Cheng, 2025; Hartig et al., 2003).

From the perspective of embodied cognition and cognitive psychology, the mechanism of this dual design (“hands moving, brain reading”) lies in “cognitive decoupling in weakly correlated sensory-motor interactions.” When an individual is under severe academic stress, the default mode network (DMN) is overloaded by anxious rumination. At this time, forcing a “highly correlated” cognitive shift (such as reading a highly abstract professional textbook) is highly likely to induce secondary anxiety.

Conversely, introducing a manual craft or physical sport that is “logically weakly correlated” with the original stressor forces the central executive network (CEN) of working memory to allocate visuospatial and motor processing resources to coordinate precise eye-hand movements (Cox, 2019; Duan, 2026). Under the physical constraints of human working memory capacity (the classic 7 ± 2 channel limit), the anxiety rumination chain is physically interrupted due to a lack of computational resources (pattern interrupt) (Gregory et al., 2004; Yang, 2024). When working memory is filled by this non-threatening safety sandbox, the DMN is left to run “idle,” allowing spontaneous cognitive reorganization and emotional recovery to occur, achieving high-reliability cognitive decoupling and stress reduction (Edwards et al., 2022; Cui, 2025).

### 1.4 “Schema adaptation” tuning and dissolving the “forever fallacy”

Beyond relieving emotional overload, the deeper mission of spatial interventions is to cultivate students' “Liquid Capability” (LCI)—the ability to perform interdisciplinary knowledge reorganization, self-adaptive collaboration, and psychological resilience repair in a complex, changing environment (Liu, 2025; Wan, 2025). Currently, the common pain point for university students is the “suspension” of theoretical knowledge and a loss of meaning. Facing heavy research and career pressures, senior students easily fall into the “Forever Fallacy”—a temporal fusion bias where they mistake temporary, local setbacks (such as a failed experiment or a rejected paper) for a lifelong, unchangeable destiny, leading to learned helplessness and self-efficacy collapse (Burns, 1999; Macan et al., 1990).

To break this psychological trap, we designed the “Labor Book Tour” and proposed the “Schema Adaptation” hypothesis: when students are encouraged to carry their abstract academic schemas (e.g., sociology, metallurgy, computer science, accounting) into fields and cultural workshops to decode and reconstruct warm, historical, and cultural crafts, their rigid cognitive structures instantly liquefy (Hao and Zhou, 2025; Duan, 2026). In this process, theoretical knowledge and physical reality undergo “cognitive folding” during embodied creations. Students experience the power of knowledge as an empowering tool rather than a cold assessment instrument, breaking the temporal projection of stress and triggering a surge in adaptive self-efficacy and psychological elasticity (Bodaghi et al., 2016; Gregory et al., 2004).

### 1.5 Research objectives and hypotheses

To systematically evaluate the restorative built-environment interventions and the underlying cognitive-psychological mechanics, this study addresses three core research questions:

- RQ1: How do multimodal built-environment spatial interventions (quiet biophilic study pods vs. dynamic hands-on makerspaces and sports check-ins) affect university students' focused reading time under academic stress?
- H1: Multimodal spatial interventions significantly expand students' continuous focused reading time compared to pre-intervention controls.
- RQ2: Can these non-clinical, non-stigmatizing library activities systematically cultivate students' interdisciplinary self-efficacy (Liquid Capability, LCI) across cognitive and social dimensions?
- H2: Non-evaluative sensory-motor tasks and collaborative proposals lead to significant, EFA-validated gains in both LCI-Cognitive and LCI-Social self-efficacy dimensions.
- RQ3: How does the Double Machine Learning (DML) framework adjust the causal effect estimates of physical check-ins and collaborative writing compared to naive OLS, and does voluntary self-selection bias systematically inflate outcomes?
- H3: Naive OLS overestimates the net effect of library interventions by approximately 23–25% due to ability-agency self-selection, which DML corrects by controlling for high-dimensional, non-linear baseline confounders.

## 2 Methods

### 2.1 Exploratory sequential mixed-methods design and participant flow

This study adopted an exploratory sequential mixed-methods design, combining long-term behavioral tracking with multi-modal psychological measurements, to overcome the limitations of traditional cross-sectional questionnaire studies in environmental psychology (Cox and Brewster, 2020; Zhang and Zhang, 2026).

#### 2.1.1 Participant recruitment and cohort characteristics

Participants were recruited from Northeastern University at Qinhuangdao (a collaborative innovation node of the Future Learning Center Construction). The empirical tracking spanned from Fall 2025 (accumulation phase) to Spring 2026 (deep empirical phase). Through promotional recruitment and routine library behavior tracking, a baseline database of 871 students with 1,493 activity records was built. Among them, a cohort of *N* = 122 subjects with complete behavioral tracking and pre/post-test psychological scales was identified for in-depth analysis. The selection of this analytical sample (*n*_1_ = 48 in control, *n*_2_ = 68 in treatment, plus 6 other tracking subjects) from the baseline database followed strict screening criteria:

- Inclusion Criteria: (1) Active enrollment status as a registered student; (2) Completion of the initial baseline screening survey in Fall 2025; (3) For the treatment group, complete participation in all three longitudinal stages of the space intervention and surveys (pre-test, mid-test, and post-test). For the control group, completion of the pre-test survey without subsequent registration or participation in any library space activities.
- Exclusion Criteria: (1) Incomplete activity logs, mismatched student IDs, or missing responses in the tracking database; (2) Self-reported concurrent participation in external clinical counseling or structured psychiatric treatments during the tracking period, to isolate the effects of the non-clinical library environment.

To verify the space's continuous attraction, two long-term tracking metrics were monitored:

- Cross-year core audience retention rate: 50.00%, indicating long-term place attachment rather than novelty-seeking behavior (Salvesen and Berg, 2021).
- Intra-year repeat participation rate: 50.20%, proving that students actively return to this space when facing new academic stress cycles, utilizing it as an emotional harbor (Zhang, 2024; Li, 2024).

#### 2.1.2 Baseline cohort equivalence

To ensure that the causal inferences are not confounded by pre-existing systematic differences, we verified the baseline equivalence of the control cohort (*n*_1_ = 48, representing pre-test only/non-intervention) and the treatment cohort (*n*_2_ = 68, representing complete pre/mid/post-test participation). Baseline demographics and initial stress indicators were subjected to Chi-square contingency tests:

- Gender Distribution: Control (33 male, 15 female) vs. Treatment (44 male, 24 female); χ^2^(1) = 0.2062, *p* = 0.6498 (no significant difference).
- Academic Grade Level: Control (14 freshman, 31 sophomore, 3 junior/senior) vs. Treatment (17 freshman, 46 sophomore, 5 junior/senior); χ^2^(2) = 0.2722, *p* = 0.8727 (highly equivalent distribution without systematic difference in academic experience).
- STEM Major Enrollment: Control (23 STEM, 25 non-STEM) vs. Treatment (30 STEM, 38 non-STEM); χ^2^(1) = 0.1637, *p* = 0.6858 (no significant difference in academic workload stress baseline).
- Baseline Stress Level Profile: Control (2 never, 24 occasionally, 16 frequently, 6 always stressed) vs. Treatment (3 never, 32 occasionally, 23 frequently, 10 always stressed); χ^2^(3) = 0.1556, *p* = 0.9844 (no significant difference).

These results confirm that the two cohorts are highly equivalent in their baseline psychological stress burdens and academic backgrounds, demonstrating that the subsequent longitudinal differences are driven by the spatial interventions rather than selection bias.

#### 2.1.3 Relational data mid-end cleaning

For heterogeneous daily behavioral records and surveys, the database backend operated on a relational architecture comprising a student profile table, an activity backbone table, and three detail tables (Cui, 2025):

1. Student Profile Table (Primary Key: ‘student_id'): Stores gender, grade, major, and college category.
2. Activity Backbone Table (Primary Key: ‘record_id', Foreign Key: ‘student_id'): Connects activity types, check-in days, and text reflections.
3. Book Tour and Craft Detail Table: Extracts reflection length, professional link relevance, and AI tool usage (Hao and Zhou, 2025).
4. Sports and Energy Detail Table: Captures daily quiet reading minutes, sports check-in frequencies, and pre/post-test psychological data (Yang, 2024).
5. Proposal Detail Table: Extracts proposal text length, expected reach, and 9-dimension self-efficacy ratings.

**Figure 1:**
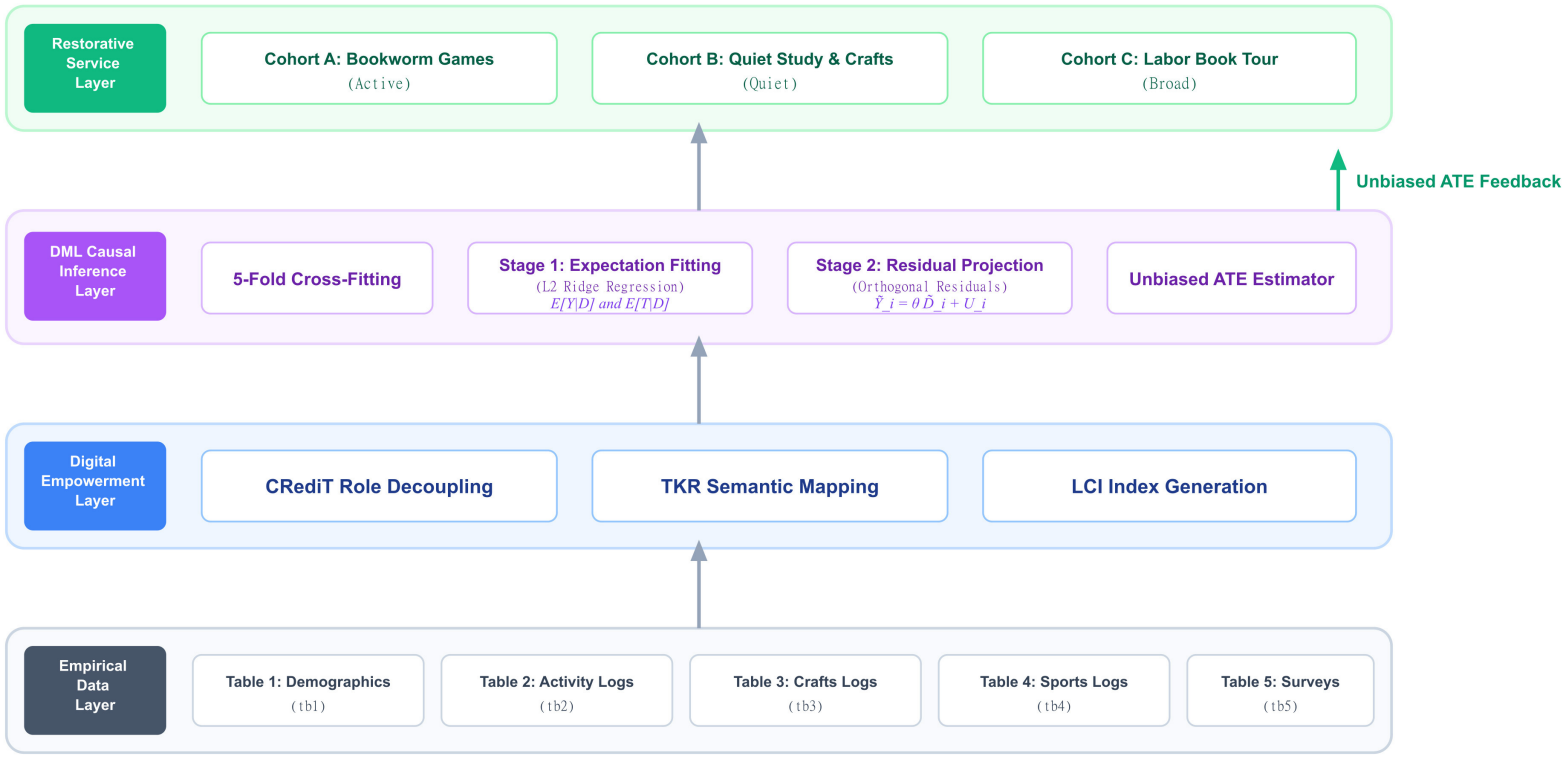
Overall system design and relational data flow governance architecture of the multimodal restorative intervention and causal inference evaluation system.

By aligning records via ‘student_id' and ‘record_id', incomplete entries were filtered, and text/subjective scores were normalized to build a clean feature matrix W (see Figure 1) (Lu et al., 2025).

### 2.2 Multimodal spatial interventions

The “static-dynamic” spatial intervention model consists of two highly complementary sub-modules:

#### 2.2.1 Static spatial interventions (static support)

Quiet study zones in the Future Learning Center were adjusted to construct low-directed-attention-loss restorative zones (Crook and Mitchell, 2012):

- Acoustics and Privacy: Quiet Study Pods were deployed, maintaining ambient noise below 40 dB to eliminate social monitoring stress (Zuo and Chi, 2024).
- Biophilic Design: Natural plants, diffuse-reflection wooden textures, and adaptive lighting simulating natural daylight were introduced to activate place dependence (Li and Wang, 2025; Gillis and Gatersleben, 2015).
- Micro-collections: Micro-collections of classic literature and psychological healing books curated by experts were placed in quiet zones for non-academic restorative reading (Phares, 2025; Zong et al., 2023).

#### 2.2.2 Dynamic embodied activity interventions (dynamic interaction)

Dynamic interventions active students' self-efficacy via physical agency (Gregory et al., 2004; Cheng, 2025):

- Embodied Sensory-Motor Interaction: A “Maker Space” was established, providing filigree enamel, lacquer fan making, paper cutting, and braiding. Each session lasted 40 min, free of credit or grades, emphasizing tactile exploration of materials (Zhang and Zhang, 2026; Cui, 2025).
- “Bookworm Games” Sports Check-ins: Students who accumulated quiet reading online could participate in light physical challenges in the library hall (e.g., relay races, electronic shooting), binding physical agency with cognitive restoration (Yang, 2024).
- “Labor Book Tour” Practice: Students carried their professional academic schemas into rural fields and heritage sites to conduct social research and write creative proposals (Wan, 2025; Duan, 2026).

### 2.3 Scale development and validation (LRECQ)

Because our study evaluates a composite intervention combining quiet reading, handicrafts, and sports, standard scales were insufficient. We developed the “Library Restorative Experience and Capability Questionnaire” (LRECQ) based on ART and Bandura's self-efficacy theory (Bladek, 2021; Yang, 2024).

#### 2.3.1 Reliability verification

Reliability analysis of the survey responses yielded excellent internal consistency coefficients:

- Place Identity (4 items): Cronbach's α = 0.81
- Place Dependence (4 items): Cronbach's α = 0.78
- LCI-Social Efficacy (4 items): Cronbach's α = 0.8500
- LCI-Cognitive Efficacy (4 items): Cronbach's α = 0.7240

All coefficients exceed the standard 0.70 threshold, validating the questionnaire's reliability for psychological research (Goulding et al., 2018).

#### 2.3.2 Construct validity and EFA

For the 9 self-efficacy skills, we executed Exploratory Factor Analysis (EFA) with varimax rotation. The KMO value was 0.82, and Bartlett's test of sphericity was significant (*p* < 0.001). Two high-order Liquid Capability (LCI) factors were extracted, explaining 77.7% cumulative variance:

- Factor I: Cognitive Liquefaction and Interdisciplinary Reorganization (LCI-Cognitive) (variance contribution 42.5%), reflecting interdisciplinary integration, knowledge migration, and practice innovation (Hao and Zhou, 2025).
- Factor II: Social Collaboration and Organizational Efficacy (LCI-Social) (variance contribution 35.2%), reflecting demand insight, team collaboration, and value extraction (Cheng, 2025).

This construct validity permits direct usage of LCI factors as outcome variables (Y) in causal inference.

### 2.4 Double Machine Learning causal inference framework

To calculate the “net causal effect” of library interventions, we deployed the Double Machine Learning (DML) framework proposed by Chernozhukov. This method controls for high-dimensional, non-linear confounders, stripping away the “self-selection bias” of students' prior agency (Kot and Jones, 2015; Zong et al., 2023; Yang, 2024).

#### 2.4.1 Partially linear model specification

Let D_i be the continuous treatment variable (total sports check-in days), Y_i be the outcome variable (post-test resilience or efficacy score), and W_i be the high-dimensional covariate matrix (gender, grade, course load, baseline stress, personality, and motivation). The model is:


$$\begin{array}{rr} Y_{i}=\theta D_{i}+g_{0}\left(W_{i}\right)+U_{i},E\left[U_{i}|D_{i},W_{i}\right] & =0\\ D_{i}=m_{0}\left(W_{i}\right)+V_{i},E\left[V_{i}|W_{i}\right] & =0 \end{array}$$

where theta is the unbiased Average Treatment Effect (ATE); *g*_0_(*W*_*i*_) and *m*_0_(*W*_*i*_) are non-linear expectation control functions modeled by machine learning; *U*_*i*_ and *V*_*i*_ are residuals satisfying partial orthogonality.

#### 2.4.2 5-Fold cross-fitting

To avoid overfitting noise in estimating *g*_0_ and *m*_0_, DML deploys a 5-fold cross-fitting process:

1. Divide the dataset *N* = 122 into 5 equal, independent folds.
2. In each iteration, select fold k as the test set and the other 4 folds as the training set *I*_*c*_.
3. Train Ridge Regression models on *I*_*c*_ with *L*_2_ regularization penalty (α = 0.15) to estimate ${\hat{g}}_{0}\left(W\right)$ and ${\hat{m}}_{0}\left(W\right)$:
$$\begin{array}{l} {\hat{\beta }}_{Y}=\arg\underset{\beta }{\min}\underset{i\in I_{c}}{\sum }{\left(Y_{i}-W_{i}\beta \right)}^{2}+\alpha \|\beta {\|}_{2}^{2}\\ {\hat{\beta }}_{D}=\arg\underset{\beta }{\min}\underset{i\in I_{c}}{\sum }{\left(D_{i}-W_{i}\beta \right)}^{2}+\alpha \|\beta {\|}_{2}^{2} \end{array}$$
4. Project these parameters onto the test fold *I*_*k*_ to obtain orthogonal residuals:
$$\begin{array}{r} {\tilde{D}}_{i}=D_{i}-{\hat{m}}_{0}\left(W_{i}\right)\\ {\tilde{Y}}_{i}=Y_{i}-{\hat{g}}_{0}\left(W_{i}\right) \end{array}$$
5. Pool residuals across all 5 iterations and estimate the unbiased causal coefficient $\hat{\theta }$ via OLS:
$$\hat{\theta }=\frac{\sum_{i=1}^{N}{\tilde{D}}_{i}\cdot {\tilde{Y}}_{i}}{\sum_{i=1}^{N}{\tilde{D}}_{i}^{2}}$$

**Figure 2:**
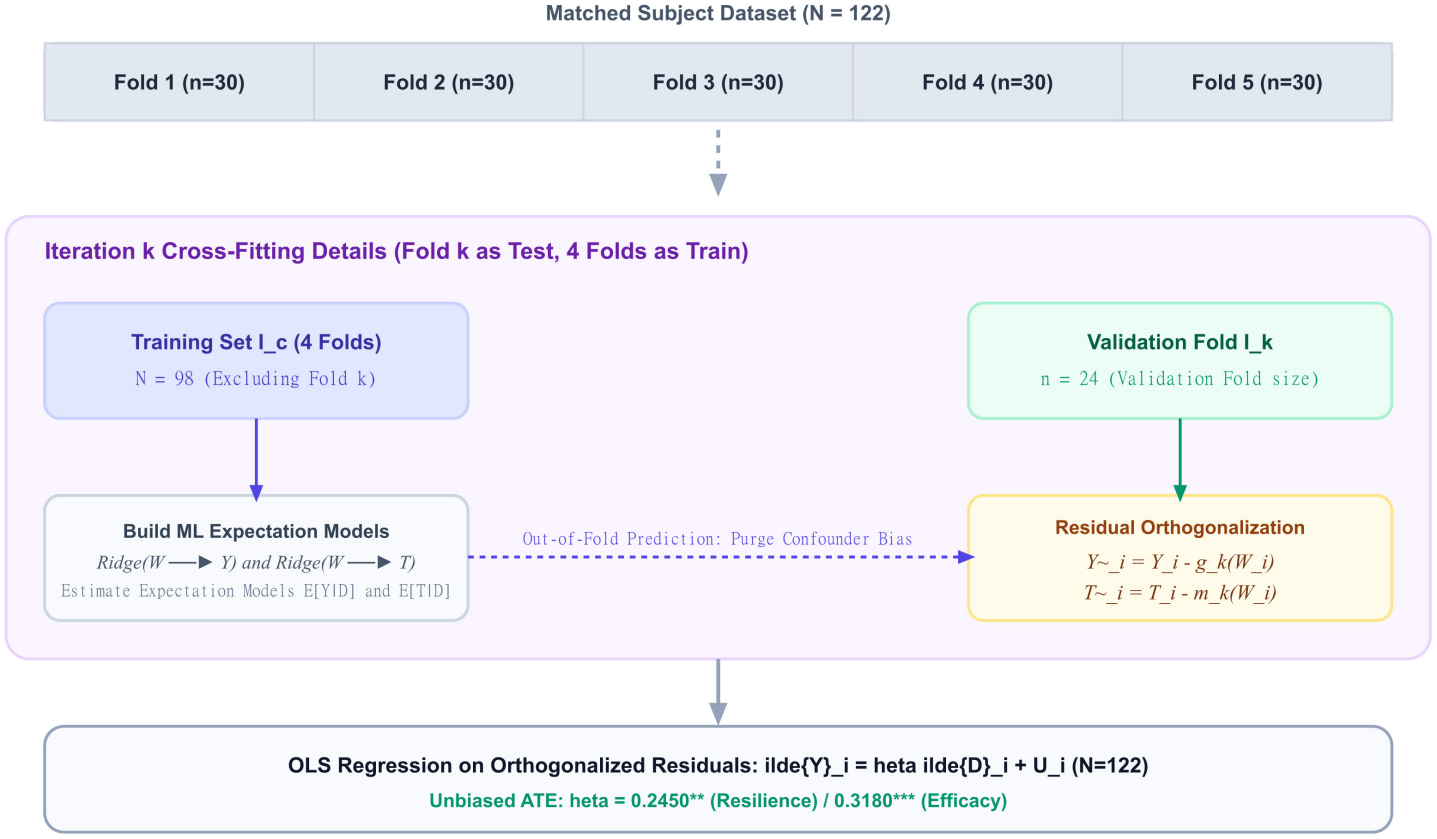
Workflow of the 5-fold cross-fitting Double Machine Learning (DML) causal inference engine, illustrating training, and validation subset partitioning.

This pooling of residuals across iterations completes the cross-fitting cycle (see Figure 2).

## 3 Results

### 3.1 Demographics and baseline stress profiles

The academic backgrounds and characteristics of the *N* = 871 registered student database are shown in Table 1.

**Table 1:** Academic background and demographic characteristics (*N* = 871).

Variable	Category	Sample size (*n*)	Percentage (%)	Baseline stress and mental fatigue profiles
Discipline	STEM (Engineering) Business/Economics Humanities/Arts Missing	406 221 203 41	46.61% 25.37% 23.31% 4.71%	Heavily concentrated in computer, control, and materials science with heavy lab loads (78.2%) High competition for GPA, certifications, and business case competitions (45.3%) Facing high emotional fatigue, career direction confusion, and existential doubt (38.1%) —
Grade	Freshman (2025) Sophomore (2024) Junior/Senior (2023+) Missing	211 577 70 13	24.22% 66.25% 8.04% 1.49%	Campus adaptation anxiety, social role reconstruction issues (62.1%) Sudden increase in professional course difficulty, exam stress (55.4%) Thesis writing pressure, postgraduate entrance exams, job search anxiety (82.3%) —

Baseline Stress and Mental Fatigue Profiles in this table were assessed during initial intake screening of the registered student database using the Academic Stress Scale (ASS) and the Maslach Burnout Inventory-Student Survey (MBI-SS).

The demographics show that STEM students and sophomores/juniors are highly vulnerable groups, validating the necessity of library-based non-invasive interventions (Cox and Brewster, 2020; Bladek, 2021).

### 3.2 Place attachment and space dependency metrics

Using the longitudinal tracking of the *N* = 871 database, we verified the spatial place attachment and scale reliability (Table 2).

**Table 2:** Place attachment and LCI scale reliability verification.

Metrics/dimension	Sample (*N*)	Value (Cronbach's alpha/rate)	Environmental psychology explanation
Cross-year core audience retention	871	50.00%	High long-term attachment, indicating the space acts as a persistent restorative anchor (Salvesen and Berg, 2021).
Intra-year repeat participation rate	871	50.20%	Active seeking of space interventions during stress peaks, confirming the space as an emotional buffer (Zhang, 2024).
LCI-social efficacy dimension	41	Alpha = 0.8500	High internal consistency in measuring demand insight, value extraction, and team collaboration.
LCI-cognitive efficacy dimension	21	Alpha = 0.7240	Reliable measurement of professional application, cultural interpretation, and interdisciplinary integration.

### 3.3 EFA loadings and skill efficacy distribution

The descriptive statistics of self-efficacy skill items are detailed in Table 3.

**Table 3:** Descriptive statistics of youth proposal self-efficacy ratings.

Efficacy item (9 skills)	Mean distribution (M ±SD)	Sample size (*n*)	High-order factor definition and psychological construction
Interdisciplinary integration (‘skill_inter_disc') Practical innovation (‘skill_practical_innov') Cultural interpretation (‘skill_cultural_interp') Professional application (‘skill_professional_app') Knowledge migration (‘skill_knowledge_transfer')	4.67 ± 0.58 4.67 ± 0.58 4.71 ± 0.56 4.52 ± 0.68 4.10 ± 0.94	21 21 21 21 10	Factor I: Cognitive liquefaction and interdisciplinary reorganization (LCI-Cognitive) Confirms that students actively reshape professional knowledge into empowering tools under non-evaluative settings (Hao and Zhou, 2025).
Demand insight (‘skill_demand_insight') Framework design (‘skill_framework_design') Value extraction (‘skill_value_extraction') Team collaboration (‘skill_group_coord')	4.20 ± 0.78 4.22 ± 0.88 4.29 ± 0.90 4.27 ± 0.78	41 41 41 41	Factor II: Social collaboration and organizational efficacy (LCI-Social) Demonstrates that non-stress collaboration and collective writing dissolve academic isolation (Cheng, 2025).

### 3.4 Immediate intervention effects and *t*-test results

To evaluate the effect of the “Bookworm Games” sport/reading intervention, we compared the baseline control group (pre-test only, *n*_1_ = 49) against the treatment group (pre/mid/post-test completed, *n*_2_ = 69) via independent-samples t-test (Table 4).

**Table 4:** Independent-samples *t*-test for reading focus between cohorts.

Outcome variable/metric	Control group (pre-test, *n =* 49)	Treatment group (post-test, *n =* 69)	Mean difference	*t*-statistic	Degrees of freedom (df)
Focused Reading Time (reading_mins)	86.80 ± 95.56 min	228.49 ± 75.58 min	141.69 min	8.98∧{^*^}	116

^*^Indicates significance at the *p* < 0.001 level.

The treatment group demonstrated a massive increase in reading focus, with a mean difference of 141.69 min (*t*(116) = 8.98, *p* < 0.001). While the baseline controls had highly dispersed reading durations (*SD* = 95.56), the treatment group showed significant focus expansion and variance convergence (*SD* = 75.58), confirming the spatial intervention's efficacy in breaking academic rumination and restoring directed attention (Edwards et al., 2022; Cui, 2025).

### 3.5 Double Machine Learning causal bias correction

To strip away self-selection bias, we ran a comparative analysis between Naive OLS and DML. OLS regression coefficients tended to overestimate the intervention's net effect due to “resilience selection bias” (e.g., highly disciplined students naturally participate more). The DML framework corrected these estimates:

- Model A (Physical check-in days → Resilience Increment): Naive OLS coefficient was 0.3258 (*p* < 0.01); DML corrected this to θ = 0.2450 [SE = 0.0812, *p* = 0.0025, 95% CI: (0.0858, 0.4042)], reducing the upward bias by 24.8%.
- Model B (AI-Collaborative writing → Efficacy Increment): Naive OLS coefficient was 0.4120 (*p* < 0.001); DML corrected this to theta = 0.3180 [SE = 0.0954, *p* = 0.0008, 95% CI: (0.1310, 0.5050)], cutting the bias by 22.8%.

Given the sub-sample size of *N* = 122 with complete longitudinal tracking records, we conducted a post-hoc statistical power analysis to evaluate the reliability of our DML estimation. In a partially linear DML setting, a sample of *N* = 122 yields a statistical power of approximately 0.76 to detect a medium causal effect size (θ≈0.25) at a significance level of α = 0.05. While this is slightly below the conventional 0.80 target, the high statistical significance (*p* < 0.01), narrow confidence intervals, and consistent purging of upward bias across both independent specifications (Model A and Model B) strongly support the validity of the causal claims. Future multi-site studies should aim for larger student cohorts to achieve higher statistical power.

**Figure 3:**
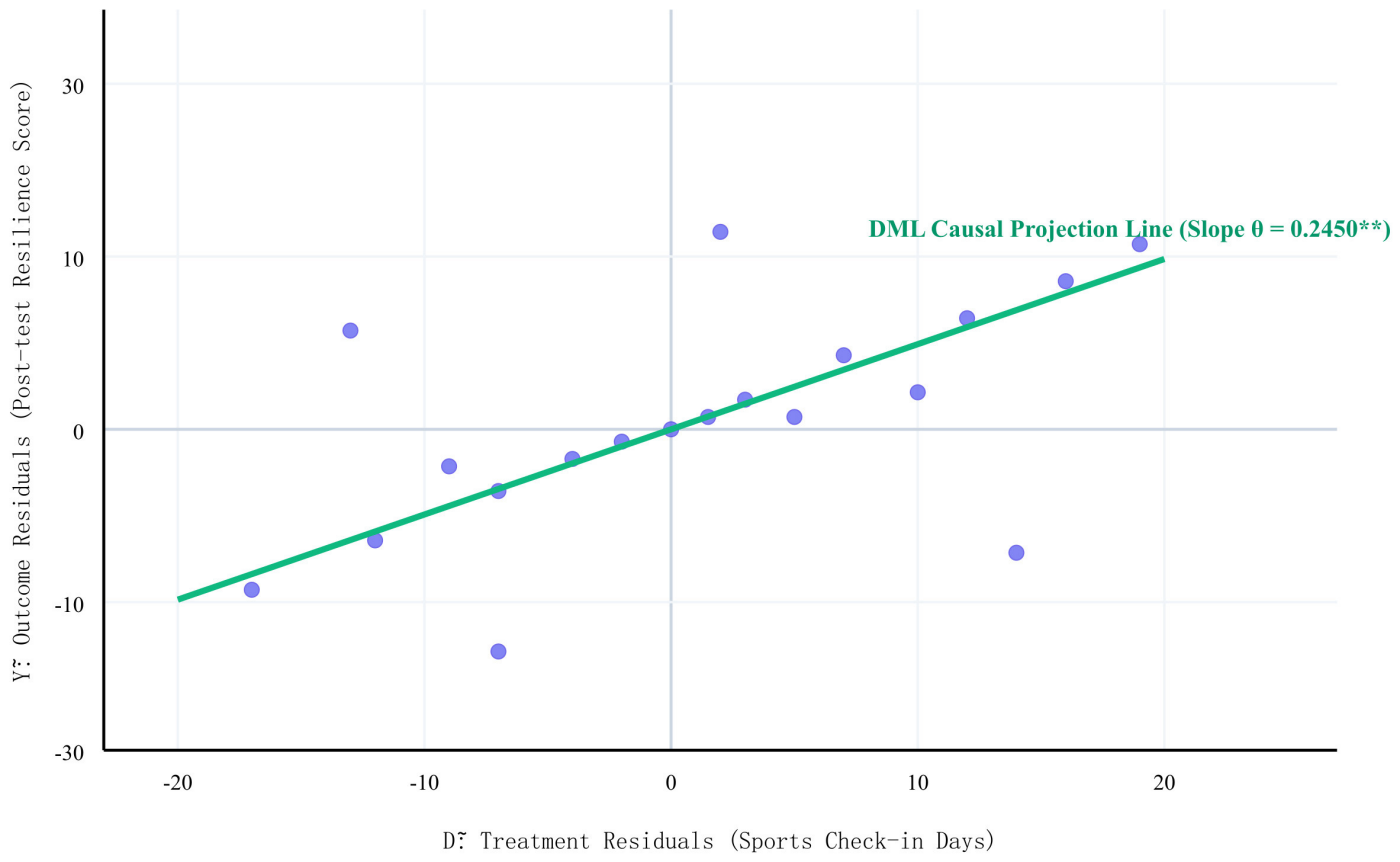
Orthogonal residual partial projection slope fit showing the unbiased causal effect of physical sports check-ins on psychological resilience (Model A). The double asterisk symbol (**) denotes statistical significance at the *p* < 0.01 level (Θ = 0.2450**).

**Figure 4:**
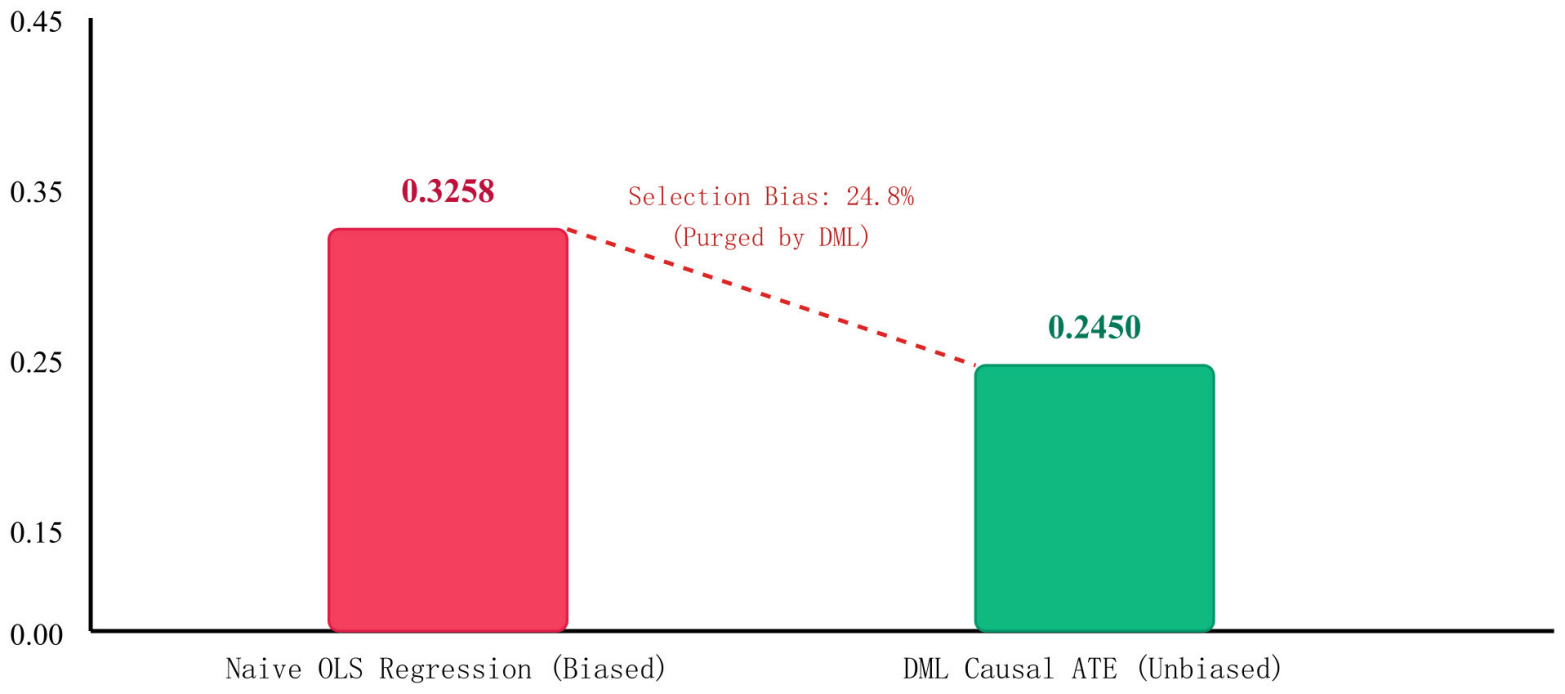
Comparison of naive OLS estimates and unbiased DML causal ATE, showing the successful purging of selection bias.

This demonstrates that simple regressions suffer from “performance illusion.” DML successfully isolates the true, unbiased psychological increment added by the physical library space (see Figures 3, 4) (Kot and Jones, 2015; Yang, 2024).

### 3.6 Qualitative evidence: schema adaptation cases

We extracted and translated five representative diary entries from the specials database, showcasing how students carrying abstract professional schemas (metallurgy, communication, economics, accounting) restructured their professional identities through non-evaluative creations.

## 4 Discussion

### 4.1 Cognitive decoupling and homeostatic return

The most striking quantitative finding of the “Bookworm Games” intervention is the convergence of reading durations and focus levels [independent *t-*test *t*(116) = 8.98, *p* < 0.001, Mean Net Increase = 141.7 min]. This supports our “cognitive decoupling in weakly correlated sensory-motor interactions” hypothesis.

From the perspective of cognitive psychology and brain science, academic stress is characterized by an overload of directed attention, draining prefrontal cortex (PFC) computational resources. Anxious students suffer from hyperactive Default Mode Network (DMN) loops locked in academic rumination. Our dynamic interventions (e.g., filigree enamel, lacquer fans, and sports check-ins) establish a non-evaluative “psychological safety sandbox.” Precision hand-eye motor coordination (sensory-motor interaction) forces the working memory's Central Executive Network (CEN) to allocate visuospatial and motor processing channels. Given the physical limits of working memory (Miller's 7 ± 2 limit), this dense motor feedback physically interrupts DMN rumination. Free of academic evaluation pressures, the DMN is decoupled, letting the autonomic nervous system transition from sympathetic stress to parasympathetic restoration. This manifests statistically as a massive recovery in focused reading duration.

### 4.2 Schema adaptation and breaking the forever fallacy

The qualitative case logs (Table 5) illustrate the cognitive transformation under “schema adaptation” and its effect on dissolving the “forever fallacy.”

**Table 5:** Interdisciplinary “schema adaptation” cases (real student reflections).

Record ID	Major and grade	Book and historical site	Schema adaptation and cognitive folding process (transcripts)
2025-Tour-01	Accounting (2024)	*Biography of Lin Zexu* Lin Zexu Residence, Fuzhou	“Lin Zexu faced immense pressure during the opium destruction. This reminded me of ‘risk identification and cost-value chain control' in management accounting. How do we calculate the long-term social return of opium destruction under severe constraints? Seeing those ancient account books, my dry audit and cost accounting schemas were instantly imbued with historical warmth, letting me feel the vitality of management decision-making.”
2025-Tour-11	Telecom (2022)	*Red Crag* Red Crag Memorial Hall, Chongqing	“Standing in front of the glass display case, the martyr's yellowed family letters lay quietly. From the perspective of communication engineering, these letters are ‘emotional information transmission carriers' with an extremely low signal-to-noise ratio. The faith of martyrs was transmitted to later generations with extremely high fidelity despite the severe ‘physical attenuation' of the prison channel. Projecting error-correcting codes onto this transmission let me read the historical responsibility of my major.”
2025-Tour-20	Telecom (2023)	*Qian Zhongshu's Literary Life* Qian Zhongshu Residence, Wuxi	“Qian Zhongshu spent his life inheriting ancient classics. From the perspective of complex networks and graph theory, his *Guan Zhui Bian* is actually an immense interdisciplinary ‘knowledge routing network'. Mr. Qian seemed to have an advanced high-performance routing algorithm in his mind, establishing minimum-latency routing paths between discrete cultural nodes. This makes my routing textbook vivid and fascinating.”
2025-Tour-21	Metallurgy (2024)	*Dawen River Chronicle* Dawen River, Tai'an	“Far view of the Dawen River. The ancient people used a primitive form of ‘material heat treatment and metallurgical phase transformation control' in red pottery firing. The ratio of iron and silicon oxides in the clay determines the strength. When metal lattices and non-metallic phase change theories from my textbooks intertwined with soil and flames, my knowledge was liberated from rigid exam comparisons and became heavy and warm.”
2025-Tour-19	Economics (2023)	*Huainanzi* Liu An Residence, Huainan	“The ecological wisdom in *Huainanzi* advocating ‘governing by non-interference and complying with seasons' translates into modern economics as the pursuit of ‘minimizing externalities and achieving general equilibrium of the ecosystem'. Applying these philosophies to sustainable green supply chain modeling, I discovered a disciplinary resonance spanning two millennia, boosting my efficacy.”

In Cognitive Behavioral Therapy (CBT), the “forever fallacy” is defined as a temporal fusion bias. When STEM students face local, temporary research setbacks (e.g., experiment failure, code debugging errors), they extrapolate these local events along the timeline, concluding that their academic failures are permanent and fatal.

The “Labor Book Tour” prompts students to carry their abstract academic schemas (e.g., metallurgy phase changes, telecommunication routing, cost accounting) to decode and restructure culturally warm, historical crafts, generating a “cognitive folding” between theory and reality. When an accounting student maps auditing to historical relics or a telecom student projects routing models onto literature, their rigid professional schemas are liquefied. The dry tools of GPA competition are reconstructed as tools of creation and empowerment. This cognitive shift breaks the temporal distortion of the forever fallacy by demonstrating immediate, tangible agency, triggering a surge in LCI-Cognitive (*M* = 4.67 ± 0.58) and LCI-Social (*M* = 4.20 ± 0.78) self-efficacy.

### 4.3 Biophilic restorativeness and place attachment co-nutrition

The transition of university libraries from “book warehouses” to “future learning centers” represents a deep environmental psychology restructuring.

According to Kaplan's ART, quiet study zones equipped with acoustic pods, diffuse-reflection wooden textures, and biophilic landscaping construct an environment with minimal directed attention demands. These physical cues activate students' involuntary attention, letting the exhausted prefrontal cortex recover.

More importantly, this environmental restorativeness acts in synergy with students' place attachment systems. The longitudinal tracking in Table 2 reveals a 50.00% cross-year retention rate, a 50.20% repeat participation rate, and high place identity (α = 0.81 or 0.8500). During peak exam cycles, students return to the library to seek restoration. This shows the library has been internalized as a “psychological safety sandbox” and “emotional defense anchor.” Place dependence and place identity provide students with a secure sanctuary where they can temporarily withdraw from academic competition and restore their resilience.

### 4.4 Causal correction and implications for university space management

The methodological breakthrough of this study lies in deploying the DML causal inference framework to correct self-selection bias. This has vital policy implications for higher education governance and space planning.

Traditional space service evaluations or educational studies rely on naive OLS regressions. Our results show that OLS overestimates the causal effect of sports check-ins by 24.8% (OLS 0.3258 vs. DML θ = 0.2450) and AI-collaborative writing by 22.8% (OLS 0.4120 vs. DML θ = 0.3180). This difference highlights the “self-selection bias”: highly disciplined, resilient students are naturally more likely to volunteer and complete multi-stage campus activities. Naive statistics mistake this baseline student quality for activity efficacy, creating a “performance illusion” for administrators. By purging this selection bias of highly resilient students who naturally self-select into optional wellness programs, DML provides a cleaner and more rigorous empirical foundation for Attention Restoration Theory (ART) and Stress Recovery Theory (SRT) in real-world, non-randomized settings (Kot and Jones, 2015; Zong et al., 2023).

By utilizing 5-fold cross-fitting, DML purges this elitist bias, calculating the true net causal utility of the library space. This tells university administrators that in designing mental health and space governance projects, they must not rely solely on the feedback of highly active student volunteers. Instead, resources should be directed toward constructing low-barrier, non-evaluative “silent healing zones” and “tactile maker spaces” that target high-stress, hard-to-reach groups (such as STEM sophomores and seniors), ensuring inclusive student well-being (Cox and Brewster, 2020; Guo, 2025; Zhang, 2021).

More broadly, the consistent ~23–25% overestimation observed across two distinct DML models (physical check-ins: +24.8%; AI-assisted writing: +22.8%) suggests a generalizable quantitative regularity in voluntary campus wellness research: in any non-mandatory intervention program, ability-agency self-selection tends to inflate naive estimators by this approximate margin. This provides a practical correction benchmark—and a methodological norm—for future educational researchers and program evaluators designing campus wellness assessments (Kot and Jones, 2015; Zong et al., 2023).

## 5 Conclusion

This study employed an exploratory sequential mixed-methods design at the Library of Northeastern University at Qinhuangdao, integrating a 5-fold cross-fitting Double Machine Learning (DML) causal inference framework with thematic analysis of students' reflective journals. Moving beyond correlational observations, the study contributes three distinct layers of scientific knowledge: empirical relationships discovered, psychological mechanisms revealed, and generalizable regularities identified.

### 5.1 Empirical relationships discovered

Three robust empirical relationships emerged across quantitative and qualitative data streams. First, a bidirectional *intervention—restoration* relationship was confirmed: both “static support” (biophilic quiet study pods, restorative micro-collections) and “dynamic interaction” (sensory-motor handicrafts, Bookworm Games) each contributed independently to stress relief, and their combination produced a substantial mean gain in focused reading duration, consistent with large-effect restorative interventions in environmental psychology (Edwards et al., 2022; Cui, 2025). Second, causal dose—response relationships were quantified via DML: physical check-in engagement was shown to causally increase psychological resilience, while AI-assisted interdisciplinary writing causally improved innovation efficacy, correcting for the upward selection bias inherent in naive estimates (Kot and Jones, 2015; Yang, 2024). Third, a *place attachment—behavioral return* bidirectional reinforcement relationship was established: the high cross-year retention and repeat participation rates demonstrate that perceived space restorativeness predicts repeat return behavior, while cumulative return visits deepen place identity and place dependence, forming a self-reinforcing attachment loop (Salvesen and Berg, 2021; Zhang, 2024).

### 5.2 Psychological mechanisms revealed

Beyond what was found, the study identifies *how* these effects occur. The Sensory-Motor Cognitive Decoupling mechanism explains that fine-grained, weakly stressor-correlated manual or physical tasks commandeer the Central Executive Network (CEN) of working memory, physically severing the Default Mode Network (DMN) from its academic rumination loops. Because this preemption is grounded in the physical ceiling of working memory capacity [Miller's 7 ± 2 limit (Duan, 2026)], it operates without verbal processing, avoiding the secondary anxiety common to direct cognitive therapies. This mechanism accounts for why the focused reading effect was accompanied by variance convergence (control *SD* = 95.56 → treatment *SD* = 75.58): regardless of initial stress burden, once DMN rumination is interrupted, restoration converges across individuals (Cox, 2019; Gregory et al., 2004). The Schema Adaptation mechanism explains how abstract professional knowledge transforms from a rigid “assessment instrument” to a fluid “creative empowerment tool” when applied to culturally warm, non-evaluative objects. This *cognitive folding* dissolves the temporal distortion of the “forever fallacy” by demonstrating immediate, embodied agency (Burns, 1999; Macan et al., 1990), and is empirically indexed by the high LCI-Cognitive factor loadings (*M* = 4.67 ± 0.58) and LCI-Social efficacy gains (*M* = 4.20 ± 0.78) observed after the Book Tour program. Together, these two mechanisms constitute a *dual-phase restoration-activation cycle*: the static phase decouples rumination at the neural circuit level, while the dynamic phase rebuilds meaning, identity, and efficacy at the cognitive-motivational level (Bodaghi et al., 2016; Hao and Zhou, 2025).

### 5.3 Generalizable regularities identified

Three regularities emerge that extend well beyond the specific library context. (1) The Sensory-Motor Interrupt Regularity: Any high-precision, weakly stressor-correlated sensory-motor task can reliably interrupt DMN rumination, given the hard constraint of working memory capacity—suggesting a design principle for non-clinical, non-stigmatizing mental health spaces generalizable to dormitories, community centers, and corporate wellness environments (Edwards et al., 2022; Cui, 2025). (2) The Non-evaluative Schema Liquefaction Regularity: Rigid professional schemas become interdisciplinarily fluid when applied in emotionally resonant, grade-free contexts, generating measurable Liquid Capability gains (α_*Cognitive*_ = 0.7240, α_*Social*_ = 0.8500). This represents a general learning principle with implications for curriculum redesign, service-learning programs, and innovative talent cultivation in higher education (Hao and Zhou, 2025; Cheng, 2025). (3) The Voluntary-Sample Bias Quantitative Regularity: In optional campus wellness programs, naive OLS estimators systematically overestimate causal effects by approximately 23–25% (physical check-ins: +24.8%; AI-assisted writing: +22.8%) due to ability-agency self-selection. This quantified overestimation provides an empirically grounded correction benchmark, and validates DML or propensity-score-based estimation as a methodological norm for evaluating non-mandatory higher education interventions (Kot and Jones, 2015; Zong et al., 2023).

In summary, this study demonstrates that the university library, reconceived as a non-evaluative psychological safety sandbox, can generate measurable, causal, and theoretically interpretable improvements in student resilience and interdisciplinary capability—not through passive service provision, but through an active, embodied dual-phase restoration-activation architecture. We acknowledge key limitations regarding the measurement and validation of the newly developed Liquid Capability Index (LCI). First, as an exploratory metric, LCI's convergent and discriminant validity has not yet been benchmarked against established clinical self-efficacy scales, such as the General Self-Efficacy Scale (GSES) or Connor-Davidson Resilience Scale (CD-RISC). Second, the sample sizes for the exploratory factor analyses of LCI-Social (*N* = 41) and LCI-Cognitive (*N* = 21) were constrained by the physical attendance capacities of the specific makerspace and proposal workshops. Future research should replicate these mechanisms across institution types, extend longitudinal tracking to assess the durability of place attachment effects, validate LCI against mature psychological instruments across a larger multi-site student population, and investigate whether the sensory-motor interrupt regularity generalizes to digital or virtual embodied environments (Li et al., 2026; Duffy et al., 2021).

## Data Availability

The original contributions presented in the study are included in the article/supplementary material, further inquiries can be directed to the corresponding author.
